# Practice recommendations to manage Alzheimer’s disease based on the targeted behavioral and psychological symptoms

**DOI:** 10.1192/j.eurpsy.2024.1322

**Published:** 2024-08-27

**Authors:** Y. Ouazzani Housni Touhami, E. Layoussifi, R. Benjelloun

**Affiliations:** ^1^Faculty of Medicine, Mohammed VI University of Health and Sciences; ^2^Psychiatry Department, Cheikh Khalifa International University Hospital; ^3^Psychiatry Department, Mohammed VI International University Hospital, Casablanca, Morocco

## Abstract

**Introduction:**

Behavioral and psychological symptoms (BPS) of Alzheimer’s disease, known as neuropsychiatric symptoms, involve a range of symptoms that include agitation, psychosis (hallucinations, delusions), affective symptoms (depression and anxiety), apathy, and sleep disturbances. These behavioral and psychological symptoms harm the patients’ daily lives and significantly burden their families. Managing BPS of Alzheimer’s disease requires a targeted approach focused on each symptom to achieve a better therapeutic response.

**Objectives:**

Providing practice pharmacological recommendations targeted to each of the behavioral and psychological symptoms of Alzheimer’s disease.

**Methods:**

A literature review was conducted using Medline via PubMed, Embase, PsycINFO, and Cochrane databases until September 2023.

**Results:**

There is a consensus in the literature that non-pharmacological approaches should be recommended as the first-line treatment for most behavioral and psychological symptoms of Alzheimer’s.

Second-generation antipsychotics (risperidone and olanzapine, with improved efficacy; aripiprazole and quetiapine, with better tolerance) are recommended for severe agitation states with a risk of self or hetero-aggression, as well as for persistent psychotic symptoms in Alzheimer’s disease. The benefit-risk balance of these agents must be assessed, with close monitoring of heart arrhythmias, metabolic risk, orthostatic hypotension, and extrapyramidal symptoms. The recommendations suggest tapering antipsychotics within the first three months of their prescription. Selective serotonin reuptake inhibitors (SSRIs) such as Escitalopram, Citalopram, and Sertraline can be considered a therapeutic option for persistent affective symptoms (depression and anxiety) with significant functional impairment or suicidal risk, severe apathy, or constant agitation. Minimum effective doses are recommended for Escitalopram and Citalopram due to the risk of QT interval prolongation. There is limited evidence regarding the effectiveness of benzodiazepines, mood stabilizers, cholinesterase inhibitors, and memantine for various behavioral and psychological symptoms; the benefit-risk ratio and therapeutic response do not support the prescription of these agents. Melatonin and Mirtazapine have limited benefits for sleep disturbances, while benzodiazepines, antihistamines, and antipsychotics should be avoided.

**Conclusions:**

The pharmacological approach should target a thorough clinical assessment of the psychopathological dimensions of behavioral and psychological symptoms of Alzheimer’s disease. The prescription should be based on evaluating the benefit-risk balance and adherence to literature recommendations for patient safety.

**Disclosure of Interest:**

None Declared

